# Multiple control levels of root system remodeling in arbuscular mycorrhizal symbiosis

**DOI:** 10.3389/fpls.2013.00204

**Published:** 2013-06-18

**Authors:** Caroline Gutjahr, Uta Paszkowski

**Affiliations:** ^1^Institute of Genetics, University of MunichMartinsried, Germany; ^2^Department of Plant Sciences, University of CambridgeCambridge, UK

**Keywords:** arbuscular mycorrhiza, root system architecture, lateral root, plant nutrition, symbiosis, Glomeromycota

## Abstract

In nature, the root systems of most plants develop intimate symbioses with glomeromycotan fungi that assist in the acquisition of mineral nutrients and water through uptake from the soil and direct delivery into the root cortex. Root systems are endowed with a strong, environment-responsive architectural plasticity that also manifests itself during the establishment of arbuscular mycorrhizal (AM) symbioses, predominantly in lateral root proliferation. In this review, we collect evidence for the idea that AM-induced root system remodeling is regulated at several levels: by AM fungal signaling molecules and by changes in plant nutrient status and distribution within the root system.

## INTRODUCTION

When plants made the transition from freshwater to terrestrial environments more than 400 million years ago, fundamental morphological changes were needed for the acquisition of mineral nutrients from the soil instead of from the aqueous substratum. Preceding the development of complex root systems the alliance with symbiotic fungi such as the Glomeromycetes and Mucoromycotina is believed to have greatly assisted this transition (Humphreys et al., 2010; Bidartondo et al., 2012; Field et al., 2012, and citations therein). The aseptate hyphal network of the glomeromycotan fungi functions as a mineral nutrient-transfer pipeline from the soil-exploring extraradical mycelium to the intracellularly colonized plant cell. Extensively branched tree-like fungal haustoria, the arbuscules, form within living plant cells and are the site of mineral nutrient delivery. It is widely accepted that these hyphal conduits have served mineral nutrient uptake by ancestral rootless gametophytes and continue to do so on today’s complexly rooted sporophytes. Liverworts constitute the earliest diverging plant lineage known (for recent review, see Jones and Dolan, 2012), that supports the development of arbuscular mycorrhizal (AM) symbioses with Glomeromycetes. The fungus enters via the rhizoid, develops arbuscules within the green thallus parenchyma (Russell and Bulman, 2005; Ligrone et al., 2007; Hata et al., 2010) and confers nutritional benefit to the plant host (Humphreys et al., 2010). In higher plants, arbuscules develop in root cortex cells where they deliver inorganic phosphate to the plant (Javot et al., 2007a; Yang et al., 2012). The extant ability of AM fungal species to equivalently colonize thallus parenchyma and root cortex suggests the genetic repertoire of AM fungi to ascertain a seamless adaption from ancient to newly invented organs and the two participating plant cell types to represent sufficiently similar niches for colonization. Within the highly patterned “modern” root system, arbuscular colonization is restricted to cortex cells.

Root systems consist of individual modules with different function: the shoot-born dicotyledon tap roots and monocotyledon crown roots (CRs) are mainly involved in anchorage and support whereas lateral roots mediate nutrient uptake (McCully and Canny, 1988). Root system architecture displays a high developmental plasticity in response to environmental stimuli such as nutrient and humidity levels or temperature (Lopez-Bucio et al., 2003; Osmont et al., 2007; Hodge, 2009). Importantly, among other biota AM fungi influence root system architecture, most prominently, by enhancing lateral root formation. In this review, we summarize current knowledge on the selective colonization of root types by AM fungi and its impact on root architectural changes, which we propose is regulated at multiple levels.

## NON-RANDOM AM COLONIZATION OF ROOT SYSTEMS

**FIGURE 1:**
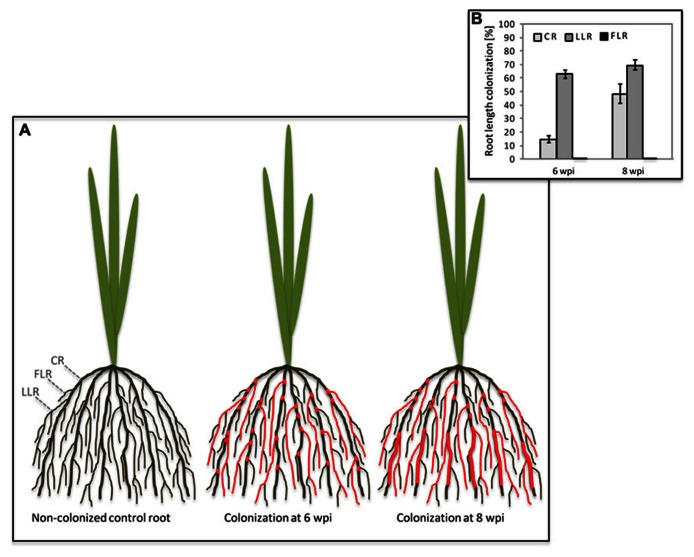
**Colonization of rice crown roots by *Glomus intraradices* upon prolonged co-cultivation.** **(A)** Schematic illustration of the rice root system with crown roots (CRs), large lateral roots (LLRs) and fine lateral roots (FLRs). Colonization of the different root types is indicated by red color. At 6 weeks post-inoculation (wpi), CR show fungal colonization at the site of lateral root emergence; at 8 wpi, the CRs are normally colonized. **(B)** Percent root length colonization of rice CR, LLR, and FLR was determined at 6 and 8 wpi with *Glomus intraradices*. Mean values and SE of four biological replicates are shown.

In both di- and monocotyledon root systems AM colonization is not evenly distributed since AM fungi preferentially colonize lateral roots and rather neglect dicotyledon primary roots or monocotyledon CRs (**Figure 1**; Hooker et al., 1992; Gutjahr et al., 2009a). Intuitively, this might be due to a higher sturdiness and lignin content in shoot-born roots with anchoring function that are more challenging to penetrate than the young expanding, and therefore softer tissue of growing lateral roots (Hepper, 1985; Amijee et al., 1993). Consistently, rigid CRs of rice are mainly colonized in patches close to lateral roots or emerging lateral root primordia (Gutjahr et al., 2009a). However, longer periods of plant co-cultivation with AM fungi increase the percentage of CR length colonized (**Figure 1**). It has been shown in maize that phosphate starvation stress leads to an increased transcription of genes involved in secondary cell wall biosynthesis (Calderon-Vazquez et al., 2008). Phosphate supply through AM fungi reduces starvation and might thus contribute to a decrease in secondary cell wall biosynthesis in CRs, thereby possibly facilitating further colonization when symbiotic phosphate transfer had conferred the effect. Also the colonization of the liverwort *Conocephalum conicum* leads to the disappearance of thallus cell wall autofluorescence at infected sites, indicating a localized decrease in cell wall phenolics (Ligrone et al., 2007). Similar to rice, also in soybean colonization was described to be particularly evident at points of lateral roots emergence. Corresponding spatial expression patterns of soluble acid invertase and sucrose synthase genes suggested an enhanced carbohydrate supply to the emerging and elongating laterals to account for this localized fungal root invasion (Blee and Anderson, 2002). Interestingly, lateral roots exhibit an increased responsiveness to AM fungal signaling molecules as evidenced by activation of a p*ENOD11*-GUS transgene in *Medicago* hairy roots (Kosuta et al., 2003). Thus they might induce the symbiotic program more swiftly and promote colonization more readily than other root types.

An unequal distribution of AM colonization is particularly evident in rice root systems, that are equipped with two types of lateral roots, the strongly colonized large lateral roots (LLRs) and the fine lateral roots (FLRs), which lack cortex tissue (Rebouillat et al., 2009), and are therefore not able to host arbuscules (Gutjahr et al., 2009a). While absence of arbuscules from FLRs was predictable, the absence of fungal hyphopodium differentiation is surprising and implies that FLRs are not recognized by the fungus (**Figure 1**; Gutjahr et al., 2009a), possibly due to differences in either their surface composition or exudation of diffusible signals. Cutin monomers have recently been shown to induce hyphopodium formation on *Medicago truncatula* roots (Wang et al., 2012). Although not yet confirmed for rice, it is an attractive possibility that FLRs release insufficient amounts of cutin or related compounds. The chemical composition of the rhizodermal surface of any plant species is not well described but there is evidence from *Arabidopsis* that it differs among root zones (Kosma et al., 2012). This is exemplified by rhizoplane bacteria, that accumulate in species-specific patterns on the *Arabidopsis* root surface (Bulgarelli et al., 2012; Lundberg et al., 2012). These patterns are likely at least in part evoked by localized chemical surface composition or differential exudation patterns. Strigolactones are constitutively exuded from higher plant roots and rhizoids of bryophytic gametophytes (Akiyama et al., 2005; Delaux et al., 2012). They induce the metabolic activity of AM fungi and provide a directional cue to guide the fungus to colonizable tissue (Parniske, 2005; Besserer et al., 2006). PDR1 (pleiotropic drug resistance protein 1), a strigolactone ATP-binding cassette (ABC)-exporter in Petunia is expressed in hypodermal passage cells of lateral roots only (Kretzschmar et al., 2012). This might explain – at least for dicotyledons – why AM fungi are firstly attracted to lateral roots. It remains an intriguing open question whether an orthologous strigolactone transporter is expressed in outer cell layers of rice FLRs.

## LATERAL ROOT INDUCTION BY AM FUNGI IS REGULATED AT MULTIPLE LEVELS

Numerous studies report root system changes in response to arbuscular mycorrhiza leading to an increased root branching and root system volume (reviewed in Hodge et al., 2009; Sukumar et al., 2013) but also reductions in root branching and length were detected (Hetrick, 1991). The basis of the observed differences is not clear but could be related to the studied plant species or the varying growth conditions. Diverging AM induced root system changes across different maize or soybean cultivars, grown under the same condition, suggested that at least part of the response is subject to genetic variation (Zhu et al., 2005; Wang et al., 2011). Although not systematically investigated an influence of the fungal genotype on the type and extend of root system remodeling can also be expected (Veresoglou et al., 2012). Yano et al. (1996) reported the induction of lateral root formation to be a highly localized response. AM inoculation of only one half of a split-root system of peanut and pigeon pea resulted in a higher number of lateral roots in the inoculated as compared to the non-inoculated half. However, systemic inhibitory or stimulatory effects on lateral root proliferation were not examined. The power of AM colonization over lateral root development was demonstrated in knock-down *Lotus japonicus* hairy root cultures of the putative transcription factor gene meristem and arbuscular mycorrhiza induced *(LjMAMI)* (Volpe et al., 2013). Here, colonization by AM fungi rescues the reduced lateral root growth phenotype and restores wild-type root system morphology. However, the most dramatic influence of AM colonization on root system architecture was found in the maize mutant *lateral rootless1* *(lrt1)* that lacks embryonic lateral roots (Hochholdinger and Feix, 1998). Inoculation with AM fungi-induced bushy lateral roots even at elevated phosphate levels (Paszkowski and Boller, 2002). Taken together these data indicate that AM fungi trigger a signaling pathway that bypasses the default lateral root developmental control exerted by MAMI and/or LRT1.

Root system architectural changes in response to AM colonization are regulated on at least two levels as evidenced by their induction prior to or after establishment of AM colonization (Berta et al., 1990, 1995; Maillet et al., 2011; Mukherjee and Ane, 2011).

### ROOT SYSTEM CHANGES IN RESPONSE TO PRE-SYMBIOTIC SIGNALING

**FIGURE 2:**
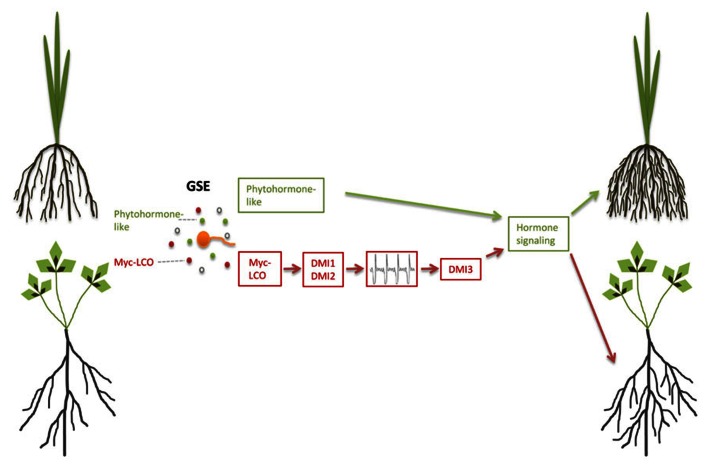
**Pre-symbiotic induction of lateral root formation in arbuscular mycorrhiza.** Germinating spore exudates (GSE) contain Myc-LCOs and possibly phytohormone-like compounds. Perception of Myc-LCOs leads to lateral root induction in *Medicago truncatula*, which requires the common symbiosis signaling components DMI1, DMI2, and DMI3 (brown pathway). The green pathway hypothesizes phytohormone-like signaling to operate either downstream or independent of common symbiosis signaling in *M. truncatula* and rice, respectively.

In the legume *M. truncatula*, germinating AM fungal spores that were separated from the root by a semipermeable membrane induced lateral root formation, indicating that diffusible signals released by these spores activate the lateral root developmental program (Olah et al., 2005). This is in agreement with the observation that the recently identified lipochitooligosaccharide Myc factors (Myc-LCOs) also induce lateral root formation in *M. truncatula* (**Figure 2**; Maillet et al., 2011). Intra-radical colonization of angiosperm roots is dependent on a signal transduction pathway, which includes Ca^2^^+^-oscillations as a second messenger and is also required for nodulation and accommodation of rhizobia and therefore named the common SYM pathway (for a recent review, see Singh and Parniske, 2012; Gutjahr and Parniske, 2013; Venkateshwaran et al., 2013). Lateral root induction by the presence of AM fungi was dependent only on *DMI1* (*POLLUX*) and *DMI2* (*SYMRK*), two genes that act upstream of Ca^2^^+^-spiking as part of the common SYM pathway (Olah et al., 2005). By contrast, Myc-LCO-mediated lateral root induction, additionally required the third common SYM gene *DMI3* (*CCamK*), that acts downstream of Ca^2^^+^-spiking (Maillet et al., 2011) and is also required for rhizobial Nod factor-mediated lateral root induction (Olah et al., 2005). This raises the question whether germinating spore exudates (GSEs) also contain diffusible signaling molecules other than Myc-LCOs that do not require DMI3, but signal through alternative components downstream of DMI1 and DMI2 to induce lateral root formation in legumes. Lateral root development might be sustained by enhanced carbon accumulation that has been described in GSE-stimulated *Lotus japonicus* roots to be dependent on CASTOR, another SYM pathway component upstream of Ca^2^^+^-spiking (Gutjahr et al., 2009b).

Remarkably, the monocot rice does not require the common SYM genes *CASTOR*, *DMI1* (*POLLUX*), and *DMI3* (*CCAMK*) for lateral root induction by GSEs (Gutjahr et al., 2009a; Mukherjee and Ane, 2011). It is intriguing whether this is due to a fundamental genetic difference between monocotyledons and dicotyledons or whether legumes, due to their specific genetic layout, that grants the development of nodules, have incorporated the common SYM pathway into a regulatory network, that directs development of all root accessory organs. Congruent with the latter hypothesis, the *Lotus japonicus* mutant *hypernodulation aberrant root formation 1* (*har1*), that hypernodulates and is hypercolonized by AM fungi, constitutively forms supernumerary lateral roots (Solaiman et al., 2000; Wopereis et al., 2000; Nishimura et al., 2002).

Lateral root formation is regulated by auxin in conjunction with other phytohormone signaling pathways (Nibau et al., 2008). Impairment of pre-symbiotic lateral root induction in hairy root culture of the auxin-resistant *diageotropica* tomato mutant suggests that Myc factor-dependent lateral root induction is similarly channeled into the auxin-controlled developmental outcome (Hanlon and Coenen, 2010). Ectomycorrhizal fungi such as *Laccaria bicolor* and *Tuber melanosporum* trigger the production of lateral roots prior to colonization through the stimulation of auxin signaling, likely due to their production and release of auxin and ethylene or other volatile compounds (Rupp et al., 1989; Karabaghli-Degron et al., 1998; Ivanchenko et al., 2008; Felten et al., 2009, Felten et al., 2010; Splivallo et al., 2009; Sukumar et al., 2013). Likewise it is possible that also AM fungi produce plant hormones such as auxin and ethylene or other volatile compounds in addition to Myc-LCOs (**Figure 2**), and this might for example explain SYM pathway-independent lateral root induction in rice, while in nodulating legumes common SYM-mediated lateral root induction might be epistatic to auxin signaling.

### ROOT SYSTEM CHANGES IN RESPONSE TO INTRA-RADICAL COLONIZATION

**FIGURE 3:**
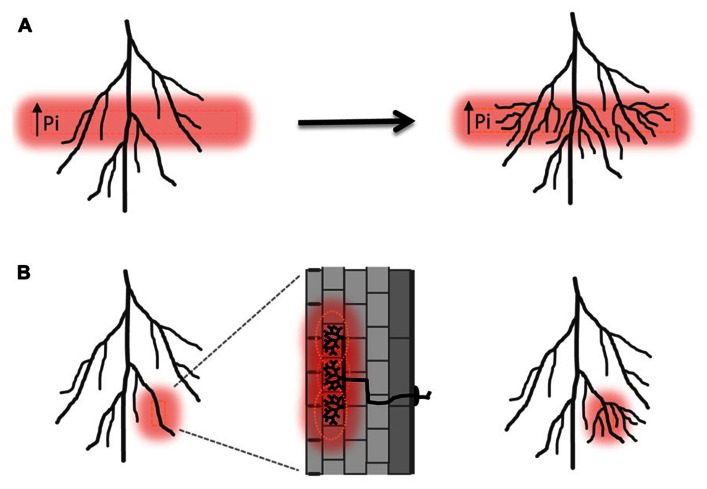
**Induction of lateral root formation in response to locally high phosphate.** **(A)** Schematic illustration of lateral root induction in high phosphate fertilized layers of the rhizosphere according to Drew (1975). **(B)** Hypothetical induction of lateral roots as a consequence of sensing a locally high concentration of phosphate within the tissue resulting from symbiotic phosphate uptake. Pi, inorganic phosphate.

Arbuscular mycorrhizal colonization preceding alterations in root system architecture has also been observed, e.g., in *Allium porrum* and *Prunus cerasifera* (Berta et al., 1990, 1995). Enhancement of lateral root formation after colonization has been related to nutritional effects. AM fungi deliver phosphate and nitrogen directly into the root cortex where the minerals are taken up by specific plant ion transporters localized in the peri-arbuscular membrane, a plant-derived membrane domain that surrounds the arbuscule branches (Harrison et al., 2002; Javot et al., 2007b; Kobae and Hata, 2010; Yang et al., 2012). The patchy distribution of AM colonization must lead to transient local increases of phosphate and/or nitrogen concentrations in the root, which may serve as a hallmark of symbiosis (**Figure 3**; Fitter, 2006). Plants can perceive localized differences in nutrient distribution also within the surrounding environment and respond with lateral root proliferation into phosphate or nitrogen-rich soil pockets (**Figure 3**; Drew, 1975; Linkohr et al., 2002). A nitrate transporter NRT1.1 has been identified in *Arabidopsis thaliana*, which acts as a nitrate transporter and sensor and triggers lateral root elongation into nitrate rich soil pockets (Remans et al., 2006). Besides nitrate it also facilitates auxin transport away from the lateral root meristem at low nitrogen conditions, leading to reduced lateral root outgrowth and elongation. In a patch of high nitrate concentration auxin transport by NRT1.1 is inhibited and auxin accumulates in lateral root tips leading to increased lateral root growth (Krouk et al., 2010). Thus NRT1.1 directly influences root system architecture via an orchestration of nitrate transport, -sensing as well as auxin transport. It will be highly interesting to determine if related mechanisms are at play in the regulation of root system architecture by mycorrhizal nutrient uptake. Mutants perturbed in mycorrhizal nutrient acquisition, e.g., defective in mycorrhiza-specific phosphate transporters such as *Medicago* PT4 or rice PT11 (Javot et al., 2007a; Yang et al., 2012), will provide a first means to study the impact of AM-mediate phosphate uptake on lateral root proliferation.

In mycorrhizal roots, the symbiotic phosphate (possibly also nitrogen) uptake pathway dominates and involves suppression of the transporter genes involved in epidermal direct uptake (Smith et al., 2003; Smith and Smith, 2011; Yang and Paszkowski, 2011). It is a currently unexplored but attractive possibility that some transport proteins belonging to the direct epidermal nutrient uptake pathway are involved in nutrient sensing similar to NRT1.1 (Krouk et al., 2010). Downregulation of their expression during the switch from the direct to the mycorrhizal nutrient uptake pathway, might inhibit sensing of the nutrient status of the surrounding soil medium, and thus alter the root system architecture response to the local soil environment thereby enhancing the influence of mycorrhizal nutrient delivery on root system architecture.

Lateral root formation can be triggered by carbon supply in the growth medium, suggesting its dependence on sufficient carbon (Jain et al., 2007; MacGregor et al., 2008). There is evidence that in the AM symbiosis fungus-delivered phosphate is traded for plant-derived carbon (Kiers et al., 2011). However, the balance of this trade can depend on the plant–fungus species combination and competition among plants that are connected via the common hyphal network (Walder et al., 2012). As long as the carbon-cost imposed by the fungus is lower than the amount of sugar transported into a given colonized part of the root system, this redirection to colonized parts of the root system could perhaps provide a mechanism by which mycorrhiza-mediated mineral nutrient uptake promotes lateral root formation (Fitter, 2006; Yang and Paszkowski, 2011). A second mechanism for liberating carbon resources might be the putative reduction of secondary cell wall biosynthesis upon phosphate starvation release (Calderon-Vazquez et al., 2008). AM colonization has been reported to induce changes in the amount of phytohormones such as cytokinins, jasmonic acid (JA), certain auxins, abscisic acid (ABA), ethylene, salicylic acid (SA), strigolactones in roots (reviewed in Hause et al., 2007; Foo et al., 2013). These phytohormones are also involved in the regulation of root system architecture (Nibau et al., 2008; Fukaki and Tasaka, 2009; Koltai, 2011). It is currently unknown in how far the changes in phytohormone levels are related to AM-induced changes in root nutrient status evoked by mineral nutrient supply via the fungus or by an increase in root carbon sink strength. Nevertheless changes in phytohormone levels might contribute to root system remodeling in response to AM colonization either independently or as part of a nutrient signaling network.

## CONCLUSIONS AND PERSPECTIVES

Plant productivity strongly depends on an appropriately adapted root system architecture for the uptake of nutrients and water under adverse soil conditions. Thus modulation of the root system architecture in response to environmental conditions is considered an important target for genetic crop improvement (de Dorlodot et al., 2007). AM fungi represent an inherent component of natural and agricultural ecosystems and influence root system architecture prior and post-colonization. It is therefore of high interest to enhance knowledge about the molecular mechanisms that underpin these morphological modulations and to elucidate the cross-talk between the two regulatory “étappes” of root system remodeling.

## Conflict of Interest Statement

The authors declare that the research was conducted in the absence of any commercial or financial relationships that could be construed as a potential conflict of interest.
